# Usefulness of fat attenuation index in postmortem CT for identifying responsible vessels in acute coronary syndromes: A case report

**DOI:** 10.1016/j.radcr.2024.08.030

**Published:** 2024-08-30

**Authors:** Tomoya Kobayashi, Junji Mochizuki, Kazuya Tashiro, Hajime Saitou, Masahiro Yoshida, Kazunori Kaga, Ryusei Inamori, Hideyuki Hayakawa, Takahisa Okuda, Yoshikazu Okamoto

**Affiliations:** a Department of Clinical Imaging, Graduate School of Medicine, Tohoku University, Miyagi, Japan; b Department of Legal Medicine, Nihon University School of Medicine, Tokyo, Japan; c Department of Radiological Technology, Minamino Cardiovascular, Tokyo, Japan; d Department of Radiological Technology, Tsukuba Medical Center Hospital, Ibaraki Japan; e Department of Radiological Imaging and Informatrics, Graduate School of Medicine, Tohoku University, Miyagi, Japan; f Department of Forensic Medicine, Tsukuba Medical Examiner's Office, Ibaraki Japan

**Keywords:** Postmortem CT, Perivascular fat attenuation index, Ischemic heart disease, Acute coronary syndrome

## Abstract

Postmortem imaging, particularly unenhanced postmortem computed tomography (PMCT), has been increasingly utilized for pathological or judicial examination as a substitute for conventional autopsy, to compensate very low autopsy rates. While unenhanced PMCT has a limitation in diagnosing acute coronary syndromes, the fat attenuation index (FAI) which is a novel imaging biomarker measured by clinical coronary CT angiography (CCTA), has been known to noninvasively detect coronary artery inflammation. We investigated the postmortem diagnostic usefulness of perivascular FAI measured by CCTA in a 61-year-old male who died suddenly after chest pain. PMCT and autopsy were conducted 92 hours after death. FAI measurement results were -57 Hounsfield units (HU) in the right coronary artery (RCA), -73 HU in the left anterior descending artery (LAD), and -64 HU in the left circumflex artery (LCX). Autopsy revealed significant stenosis in the RCA and LCX, but no significant stenosis was found in the LAD. The elevated FAI in the RCA suggested acute inflammation, which agreed with the autopsy findings. This case is the first to demonstrate effectiveness of FAI measured with PMCT for identifying the vessels responsible for acute coronary syndromes, indicating its potential in postmortem diagnosis.

## Graphical abstract

**Figure:**
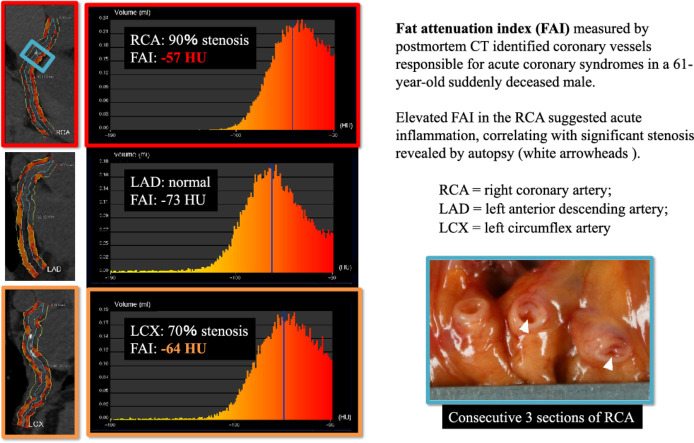

## Introduction

Postmortem imaging has been increasingly used for pathological or judicial examination as an alternative to declining autopsy rates. Unenhanced postmortem computed tomography (PMCT) has demonstrated considerable efficacy in detecting a range of hemorrhagic lesions [1]. Despite the advantages, studies indicate that the diagnostic accuracy for acute coronary syndromes (ACS) by PMCT alone remains inadequate [[2], [3], [4]]. According to Khan et al. [5], ischemic heart disease was the leading cause of death globally in 2017, affecting 126 million people and causing 9 million deaths, with prevalence expected to rise by 2030.

The fat attenuation index (FAI), which measures changes in perivascular fat on coronary CT angiography (CCTA), is a new noninvasive imaging index for detection of coronary artery inflammation [[6], [7], [8]]. The cardiovascular risk prediction study using computed tomography (CRISP-CT) by Oikonomou et al. showed cardiac mortality predictive value based on perivascular FAI when the value was elevated in the major coronary arteries [6]. Recent case studies have illustrated the utility of FAI in identifying ischemia-causing lesions, especially in cases where traditional coronary angiography fails to identify stenosis [9], emphasizing its role in diagnosing cardiac conditions including spontaneous coronary artery dissection [10].

We hypothesized that the FAI may offer a diagnostic utility in postmortem setting also, and examined a corpse that died suddenly after complaining chest pain, although PMCT alone is generally difficult to identify ACS. Herein, we report a case in which PMCT-measured FAI was useful and the results agreed with autopsy findings.

## Case report

A 61-year-old male truck driver was found in a state of cardiopulmonary arrest on a driver's cab seat approximately 30 minutes after complaining of chest pain at the office. He had a history of hypertension. Although cardiopulmonary resuscitation was performed by emergency physicians, he was pronounced dead subsequently. The corpse was kept in cold storage at 4°C after death. At approximately 92 hours after death, cardiac PMCT examination was performed with a 16-row-detector CT scanner (Aquilion Lightning, Canon Medical Systems, Otawara, Japan) at the Medical Examiner's Office before autopsy.

A dedicated workstation (AZE VirtualPlace, Canon Medical Systems, Otawara, Japan) was used for the FAI evaluation. Since measurement of vascular lumen is difficult on PMCT with no enhancement, the adventitia of the 3 main coronary artery branches on the CCTA images were manually traced along the total length at a 3-mm width using a previously reported method by Okamoto et al. [10]. Perivascular fat was defined as adipose tissue surrounding the vessel wall within a distance equal to the vessel diameter. Based on the method of Antonopoulos et al. [8], we identified perivascular FAI using the attenuation histogram of perivascular fat, within the range of -30 to -190 (Hounsfield Units: HU) that were measured by CCTA (Fig. 2). The FAI results were -57 HU in the right coronary artery (RCA), -73 HU in the left anterior descending branch (LAD) and -64 HU in the left circumflex branch (LCX) (Fig. 2), showing that the FAI of RCA is higher than that of LAD and LCX. This suggests presence of inflammation in the epicardial fat surrounding the RCA. The LCX also shows higher CT numbers than the cut-off value of -70.1 HU, which is the risk factor for coronary artery pathology [6].

**Fig. 1:**
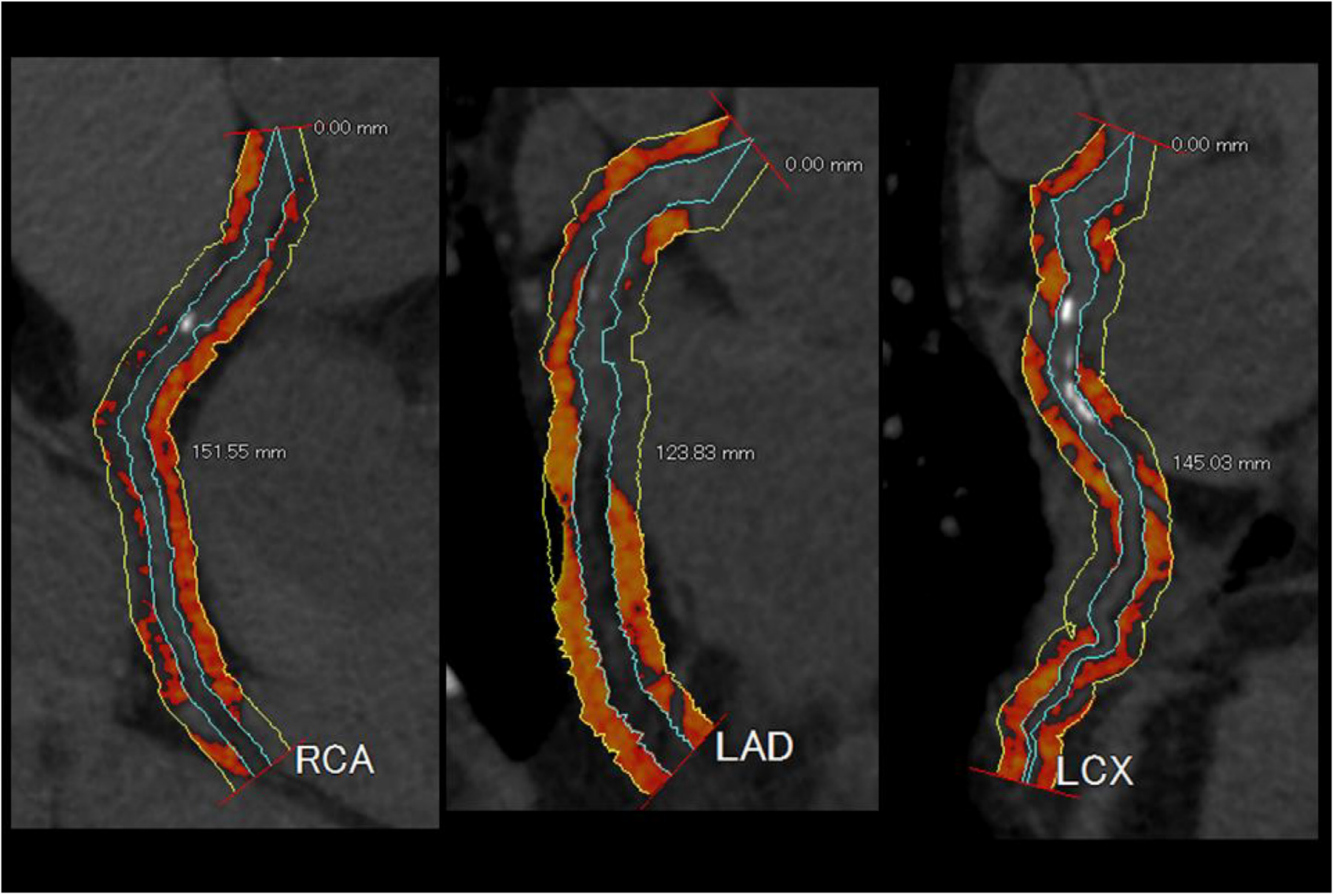
Perivascular fat attenuation index (FAI) analysis of coronary arteries. The light blue lines delineate the inner wall of the coronary arteries. The color maps show distribution of the CT numbers, where red indicates higher number and yellow indicates lower number. Abbreviations: RCA, right coronary artery; LAD, left anterior descending artery; LCX, left circumflex artery.

**Fig. 2:**
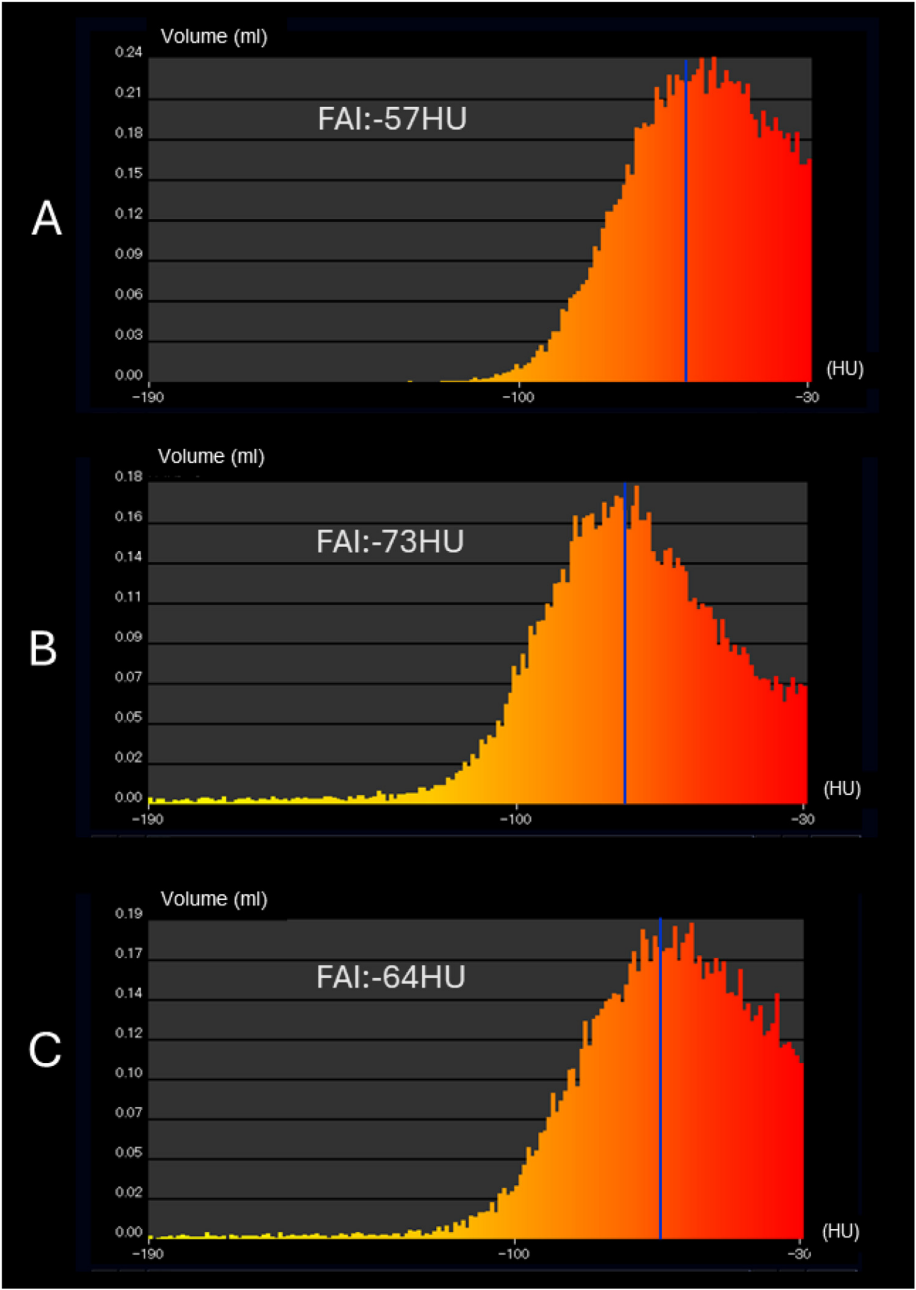
Histograms of voxel CT attenuations within the volume of interest. The horizontal line shows CT numbers (HU), ranging from -190 to – 30 HU, and the vertical line shows the volume (ml) of identical pixel values calculated by CT. (A) shows the histogram for the RCA with an FAI of -57 HU, (B) shows the histogram for the LAD with an FAI of -73 HU, and (C) shows the histogram for the LCX with an FAI of -64 HU. The color gradient from yellow to red (corresponding to Fig. 1) indicates the CT numbers within the volume of interest.

**Fig. 3:**
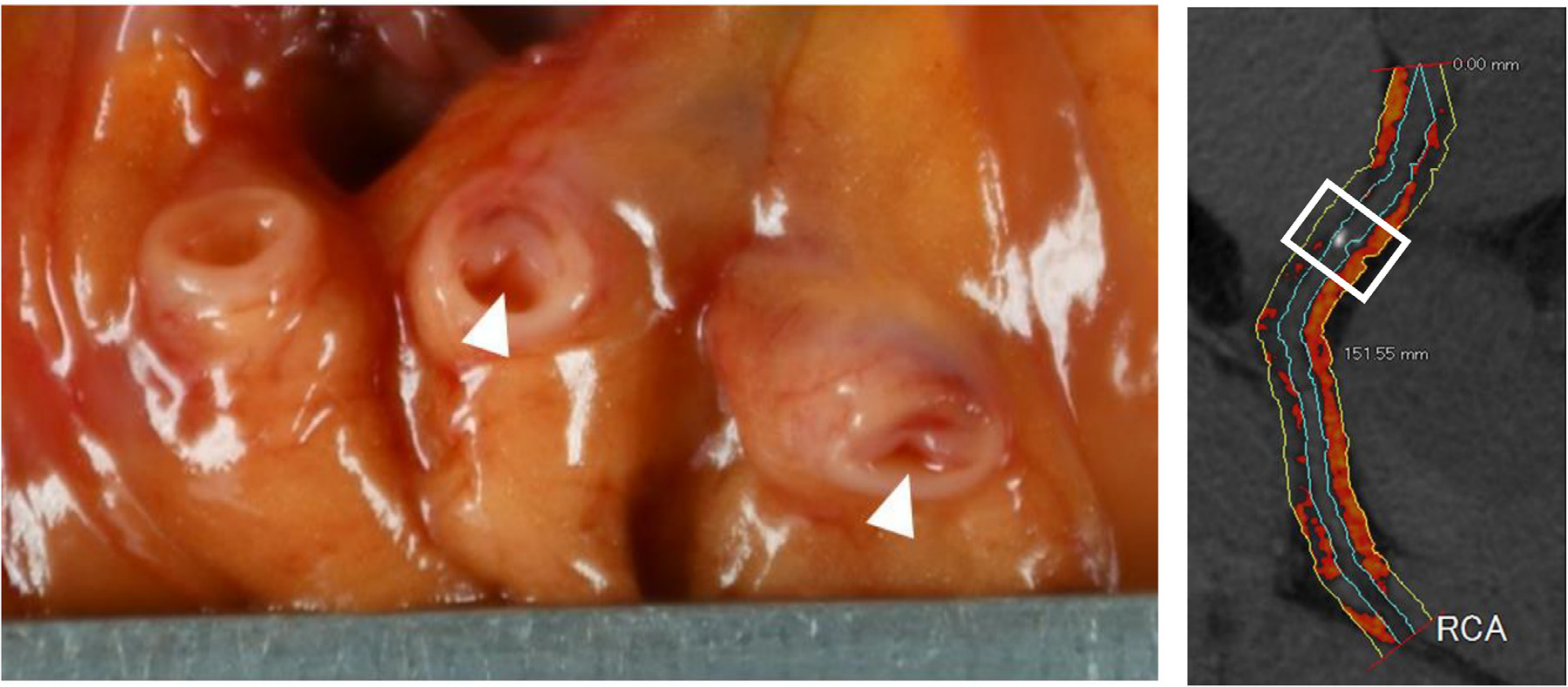
Macroscopic view of the right coronary artery. The photo shows resected and opened consecutive sections of the same RCA. The white arrowheads indicate areas of luminal stenosis (approximately 90%) with lipid plaque which was present at approximately 4.0 cm distal from the origin of the RCA, which corresponds to the region surrounded by a white rectangular line.

**Fig. 4:**
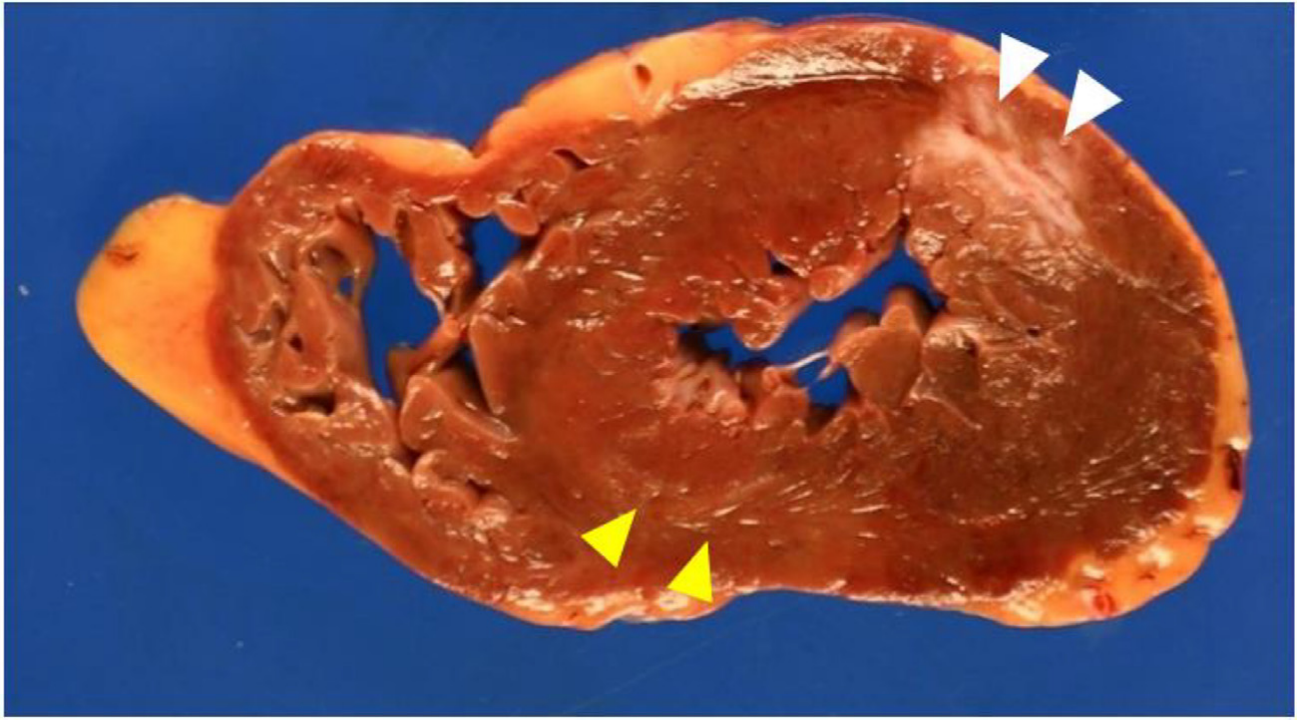
Macroscopic view of the myocardium in transverse section. The left side includes the right ventricle and the left side includes the left ventricle. The yellow arrowheads show a myocardial lesion that exhibit slightly pale coloration, suggesting areas of acute ischemic change. The white arrowheads denote areas of myocardial scarring, indicative of previous infarctions.

Autopsy revealed a right-dominant coronary artery which had around 90% luminal stenosis at approximately 4.0 cm distal from the origin of the RCA (Fig. 3); around 70% luminal stenosis at approximately 3.0 cm distal from the origin of the LCX; no significant luminal stenosis in the LAD. In the myocardium, mild changes suggestive of acute myocardial ischemia were observed from the ventricular septum to the posterior wall of the left ventricle, including contractile zone necrosis, myocardial tears, and myocardial wave-like running (Fig. 4). A whitish discoloration of approximately 1.5 × 1.5 cm in size was seen from the anterior to lateral myocardial wall, with histological evidence of fibrosis without inflammatory cell infiltration (Fig. 4, white arrowheads). The absence of stenotic lesions in the LAD led us to suspect an old myocardial infarction without cardiomyopathy or coronary artery stenosis [11]. No obvious lesions were detected in other organs by macroscopical observation and histological examination. The cause of death was determined as ischemic heart failure due to arterial sclerosis.

## Discussion

Death cause detection provides important information in improving public health issues and in criminal investigations. However, identifying the cause of death from ischemic heart disease generally requires an autopsy, as it is difficult to assess by PMCT alone [2,3]. Evaluation of pericoronary adipose tissue attenuation by CT has emerged as a highly sensitive method for assessing coronary artery inflammation, and biopsy-proven results support its validity [12]. Paracrine signaling occurred from inflammatory coronary arteries spread to perivascular fatty tissues, and inhibits local adipogenesis. This changes the composition of the perivascular fat around the inflamed artery, altering it from a lipid component to a water component, resulting in increased CT numbers [13]. Cross-sectional observational studies have also confirmed that higher CT numbers of pericoronary fat are associated with the presence of coronary arterial plaque and ruptured coronary artery lesions in ACS [6].

In our case, the cause of death was successfully identified with PMCT for the characteristic findings in the measured FAI. In particular, the lesion showed elevated FAI in RCA, which was more prominent than in LAD, where no atherosclerotic changes were observed at autopsy, suggesting that elevated FAI in the RCA reflected acute inflammation. The LCX also showed a higher FAI value (-64 HU) than the cut-off value (-70.1 HU) by CTTA [6] and the autopsy revealed 70% stenosis.

To our knowledge, this is the first case in which measured FAI values with PMCT led to the identification of the responsible vessels for acute coronary syndrome. FAI assessment with PMCT is considered useful in detecting the cause of sudden cardiac death. To further verify our conclusion, assessment with greater number of cases and various times elapsed after death are desired in the future.

## Ethical approval

This study was approved by the ethics committee of Tohoku University (approval no.2023-1-674).

## Patient consent

Written informed consent from bereaved families was obtained.

## References

[1] Takahashi N, Higuchi T, Shiotani M, Hirose Y, Shibuya H, Yamanouchi H (2012). The effectiveness of postmortem multidetector computed tomography in the detection of fatal findings related to cause of non-traumatic death in the emergency department. Eur Radiol.

[2] Inai K, Noriki S, Kinoshita K, Sakai T, Kimura H, Nishijima A (2016). Postmortem CT is more accurate than clinical diagnosis for identifying the immediate cause of death in hospitalized patients: a prospective autopsy-based study. Virchows Arch.

[3] Virtual autopsy as an alternative to traditional medical autopsy in the intensive care unit: a prospective cohort study n.d. 10.7326/0003-4819-156-2-201201170-00008 22250143

[4] Weustink AC, Hunink MGM, Van Dijke CF, Renken NS, Krestin GP, Oosterhuis JW. (2009). Minimally invasive autopsy: an alternative to conventional autopsy?. Radiology.

[5] Khan MA, Hashim MJ, Mustafa H, Baniyas MY, Al Suwaidi SKBM, AlKatheeri R (2020). Global epidemiology of ischemic heart disease: results from the global burden of disease study. Cureus.

[6] Oikonomou EK, Marwan M, Desai MY, Mancio J, Alashi A, Hutt Centeno E (2018). Non-invasive detection of coronary inflammation using computed tomography and prediction of residual cardiovascular risk (the CRISP CT study): a post-hoc analysis of prospective outcome data. Lancet North Am Ed.

[7] Mancio J, Oikonomou EK, Antoniades C. (2018). Perivascular adipose tissue and coronary atherosclerosis. Heart.

[8] Antonopoulos AS, Angelopoulos A, Tsioufis K, Antoniades C, Tousoulis D. (2022). Cardiovascular risk stratification by coronary computed tomography angiography imaging: current state-of-the-art. Eur J Prev Cardiol.

[9] Dong X, Zhu C, Li N, Shi K, Si N, Wang Y (2023). Identification of patients with acute coronary syndrome and representation of their degree of inflammation: application of pericoronary adipose tissue within different radial distances of the proximal coronary arteries. Quant Imaging Med Surg.

[10] Okamoto S, Mochizuki J, Matsumi H, Hashimoto K, Nikaido A, Hata Y. (2023). Perivascular fat attenuation index measured by coronary computed tomography angiography as a tool for assessment of ischaemia-causing lesions: a case report. BMC Cardiovasc Disord.

[11] Samaras A, Moysidis DV, Papazoglou AS, Rampidis G, Kampaktsis PN, Kouskouras K (2023). Diagnostic puzzles and cause-targeted treatment strategies in myocardial infarction with non-obstructive coronary arteries: an updated review. JCM.

[12] Antonopoulos AS, Margaritis M, Verheule S, Recalde A, Sanna F, Herdman L (2016). Mutual regulation of epicardial adipose tissue and myocardial redox state by PPAR-γ/Adiponectin Signalling. Circ Res.

[13] Antonopoulos AS, Sanna F, Sabharwal N, Thomas S, Oikonomou EK, Herdman L (2017). Detecting human coronary inflammation by imaging perivascular fat. Sci Transl Med.

